# Clostridium difficile Infection Rates During the Pandemic in New York Capital Area: A Single-Center Study

**DOI:** 10.7759/cureus.37576

**Published:** 2023-04-14

**Authors:** Spyridon Zouridis, Maheep Sangha, Paul Feustel, Seth Richter

**Affiliations:** 1 Medicine, Albany Medical Center, Albany, USA; 2 Gastroenterology and Hepatology, Albany Medical Center, Albany, USA; 3 Neuroscience and Experimental Therapeutics, Albany Medical College, Albany, USA

**Keywords:** clostridium dificle, c. diff colitis, coronavirus pandemic, covid 19, clostridium difficle infection

## Abstract

Introduction

*Clostridioides difficile (C. difficile)* colonizes the large intestine, rendering healthy individuals asymptomatic carriers of the disease. In certain instances, *C. difficile* infection (CDI) occurs. Antibiotic use remains the leading risk factor for CDI. During the coronavirus disease 2019 (COVID-19) pandemic, multiple risk and protective factors for and against CDI were identified, and as such multiple studies tried to analyze the pandemic’s overall effect on CDI incidence rates, with contradictory results. Our study’s aim is to further characterize the CDI incidence rates trends, but for a longer period of 22 months in the pandemic.

Methods

We included only adult (>18 years) patients, diagnosed with CDI during their hospitalization for the following period: January 1, 2018, to December 31, 2021. Incidence was calculated as cases per 10,000 patient days. The period identified as the COVID-19 pandemic period was the following: March 1, 2020, to December 31, 2021. All analyses were performed by an expert statistician using Minitab software (Minitab Inc., State College, Pennsylvania, United States).

Results

The mean CDI incidence rate per 10,000 patient-days was 6.86 +/-2.1. The 95% confidence interval for the CDI incidence rate prior to the pandemic was found at 5.67 +/-0.35 while the interval during the pandemic was calculated at 8.06 +/- 0.41 per 10,000 patient days. Those results reveal a statistically significant increase in CDI incidence rates during the COVID-19 era.

Conclusion

Multiple risk and protective factors for and against hospital-acquired infections (including CDI) have been identified during the unprecedented COVID-19 healthcare crisis. In the literature, there is high controversy regarding the trends of CDI incidence during the pandemic. The current study analyzed an almost two-year period into the pandemic, identifying an increase in CDI rates when compared to the pre-pandemic era.

## Introduction

*Clostridioides difficile (C. difficile)* is a Gram-positive, spore-forming, and toxin-producing bacillus. Most pathogenic strains produce two toxins, known as enterotoxin (aka Toxin A) and cytotoxic toxin (aka Toxin B). *C. difficile* colonizes the large intestine, rendering healthy individuals asymptomatic carriers of the disease. In certain instances, *C. difficile* infection (CDI) occurs. Antibiotic use, for example, which remains the leading risk factor for CDI, alters the microbial flora of the large intestine and may lead to CDI.

CDI predominant symptoms include diarrhea and colitis due to the exotoxins the strain produces. In general, it is recommended that patients with >=3 loose stools in 24 hours without clear etiology be evaluated and possibly tested for CDI. As per most recent reports, CDI affects almost 500,000 Americans annually with 29,000 having fatal outcomes [1]. During the COVID-19 pandemic, multiple risk and protective factors for and against CDI were identified, and as such multiple studies tried to analyze the pandemic’s overall effect on CDI incidence rates, with contradictory results [2-7].

Even though multiple analyses identified lower CDI rates during the pandemic, mainly attributed to the infection prevention protocols implemented during the pandemic, there were others that found an increase in CDI cases [2-4,6-8]. Plausible explanations for this increase may be the increased use of antibiotics and/or steroid or even the inpatient population profile change during the pandemic [9,10].

Our study’s aim is to further characterize the CDI incidence rates trends, but for a longer period of 22 months during the pandemic.

## Materials and methods

This was a single-center study conducted at the Albany Medical Center, Albany, New York, United States. The study period was identified as January 1, 2018, to December 31, 2021. By using the International Classification of Diseases (ICD) coding system, patients diagnosed with CDI during their hospitalization were identified.

All the adult patients (>18 years) hospitalized during the aforementioned period with a diagnosis code for CDI were included in the study. All non-adult patients were excluded from the study. Following the identification of patients with CDI from January 1, 2018, to December 31, 2021, the patients were divided into two cohorts. The first included all the patients hospitalized with CDI prior to the pandemic while the second included all the patients in the hospital who were also identified to have CDI during the COVID-19 pandemic period. The COVID-19 pandemic period was identified as March 1, 2020, to December 31, 2021. The Institutional Review Board of Albany Medical Center approved the study (approval number: 6438).

Statistical analysis

The CDI incidence was calculated as cases per 10,000 patient-days. In order to identify the patient-days for each period, we requested the daily hospital census for the period January 1, 2018, to December 31, 2021. By identifying the number of CDI cases (nominator) and by adding the hospital daily census (denominator) for specified timeframes, we were able to report our results (CDI incidence rate) as cases per 10,000 patient-days. As mentioned, in order to compare the results, the COVID-19 pandemic period was identified as March 1, 2020, to December 31, 2021. We, furthermore, categorized the data per month, in order to identify any trends in the CDI incidence rates. For the CDI incidence comparison prior to and during the pandemic, the two-sample t-test was used. CDI cases and patient-days numbers prior to and during the pandemic were also compared using the two-sample t-test. All analysis was performed by an expert statistician using Minitab software (Minitab Inc., State College, Pennsylvania, United States) [11].

## Results

In our study, the mean CDI incidence rate per 10.000 patient-days was 6.86 +/-2.1. The 95% confidence interval for the CDI incidence rate prior to the pandemic was found at 5.67 +/-0.35 per 10,000 patient days while the interval during the pandemic was calculated at 8.06 +/- 0.41 per 10,000 patient days. These results reveal a statistically significant increase in CDI incidence rates during the COVID-19 era.

**Figure 1:**
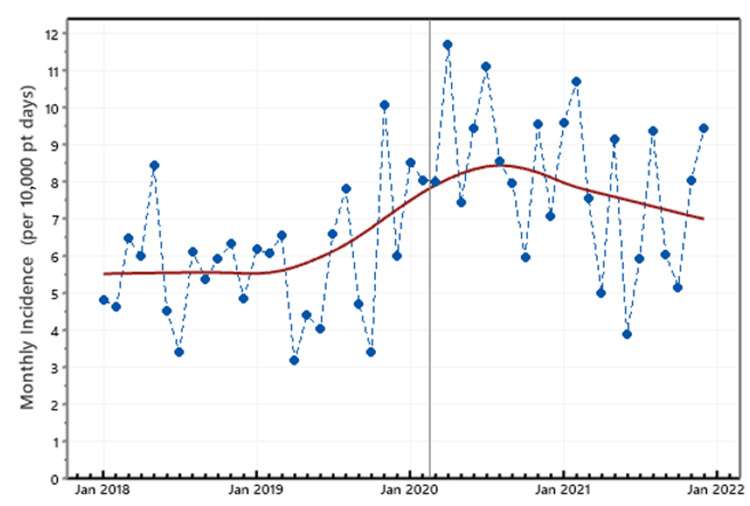
Monthly CDI incidence CDI: *Clostridioides difficile* infection; pt: patient; Jan: January

The months with the highest CDI incidence rates were May 2018, August 2019, April 2020, and February 2021 with May, August, and December 2021 following closely. The CDI incidence for those months was higher than eight cases per 10,000 patient-days and at most times higher than 10. The months with the lowest CDI incidence rates were July 2018, April and October 2019, October 2020, and June 2021 (Figure 1).

**Figure 2:**
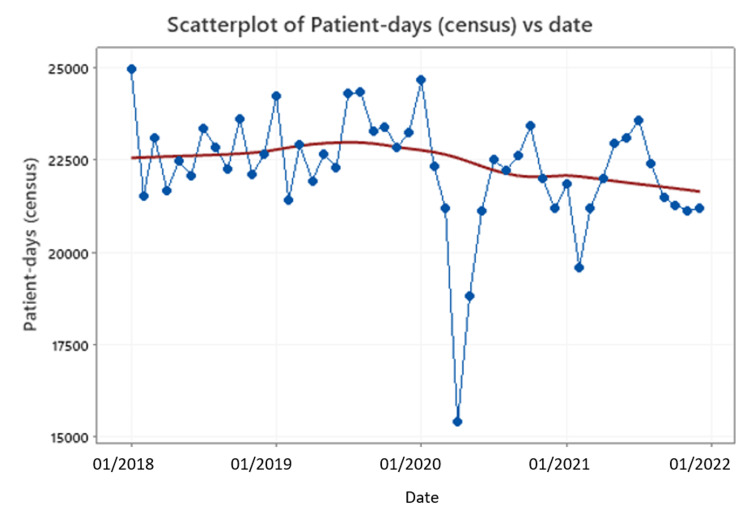
Hospital patient-days per month

**Figure 3:**
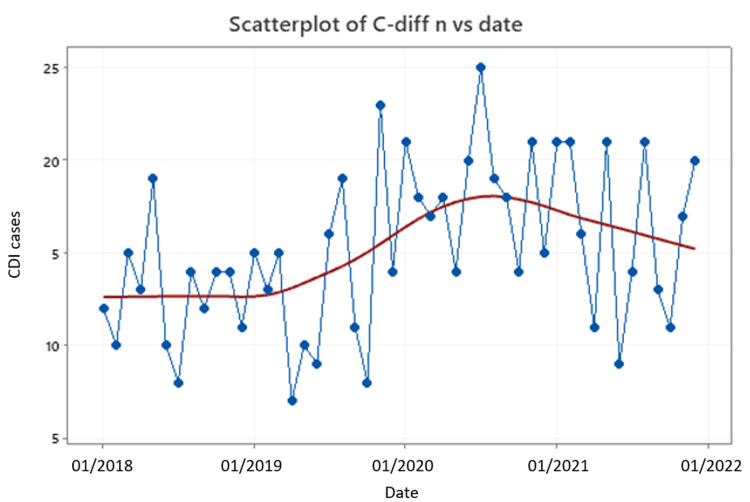
CDI cases per month CDI: *Clostridioides difficile* infection; n: number

In terms of patient-days, which was the denominator in our calculations, we noticed that in April 2020 and May 2020, the calculated numbers were much lower when compared to other months while, at the same time, the CDI cases were not significantly different than those of other months. As such, an artificially higher incidence may have been observed during those months. The same concept applies to the second pandemic wave for the month of February 2021 (Figures 2, 3). However, in general, the patient-days difference prior to and during the pandemic shows a statistically significant increase of 1260 patient-days (95%CI, 412-2108).

## Discussion

The COVID-19 pandemic was an unprecedented healthcare crisis that brought radical changes in medicine practice, especially in the inpatient setting. Implementation and strong adherence to infection prevention protocols along with mandatory use of personal protective equipment (PPE), and improved hand hygiene are only some of the changes that were adopted and are hypothesized to have positively influenced the hospital-acquired infection rates, including CDI [12-14]. However, at the same time, the inpatient population exhibited multiple risk factors for CDI development, including prolonged hospitalization, use of steroids, overuse of antibiotics, elderly age, and immunocompromised status [9,10].

During the first COVID-19 pandemic wave, multiple studies revealed lower CDI incidence rates despite unchanged testing frequency [4-6,15]. That trend was mainly attributed to better adherence to infection prevention protocols [4,6]. Moreover, other analyses on CDI incidence rates and antibiotic use during the pandemic, revealed surprising results: Despite antibiotics’ inherent risk of CDI development and the higher utilization of antibiotics during the pandemic, unchanged and/or lower CDI incidence rates were described [1,14,16,17]. Additionally, it’s noteworthy that there were also reports of a significantly higher burden on CDI patients during the pandemic despite the lower numbers [15].

On the other hand, multiple other studies showed an increase in CDI rates [2,3,7]. A recent large Canadian study involving multiple centers revealed an 11% increase in hospital-acquired CDI during the pandemic [8]. Another retrospective analysis, investigating the COVID-19 era until January 2021, identified higher CDI rates during COVID-19 peak periods [18]. Moreover, there were studies that despite not revealing any changes in CDI incidence, describe an increase in severe CDI cases [19]. Concerns have also been raised that diarrhea, which is commonly experienced by COVID-19 patients, may have been attributed to the novel virus, and as such many patients may have not been tested for CDI during the pandemic [20-22]. In our analysis, a clear increase of approximately 2.3 cases (per 10,000 patient-days) in CDI incidence was detected during the COVID-19 era. In contrast to other studies that were performed only during the first wave of the pandemic or in hospitals treating mainly COVID-19 cases, we report results for almost the first two years of the pandemic in a large tertiary care hospital that is also a referral center for a vast area in New York and neighboring states.

Other than better adherence to infection prevention protocols, plausible explanations for the lower CDI rates that were reported by many studies during the pandemic included decreased length of stay and decreased hospital occupancy [4,5,16]. Indeed, in the first two months of the pandemic in 2020, we also noticed a drop in our hospital’s patient-days. However, our study, which refers to a larger period, found significantly higher patient-days numbers when larger periods were analyzed.

In our study, we also found variability in the monthly CDI rates. In general, spring months were characterized by higher CDI incidence rates, while in summer and fall, lower rates were detected. Similar observations were reported by Rodriguez-Palacios et al. that revealed peak CDI incidence during spring and lowest during summer/autumn and attributed those results to possible contaminated food or environment [23].

The current study has some limitations including the retrospective nature of the study and data analysis from a single center. Moreover, since the study analyzed only the rates of CDI without taking into consideration any protective or risk factors for the patients identified, further studies are needed to elucidate how the factors described may have contributed to the results seen in this and other analyses.

## Conclusions

The current analysis detected a clear CDI incidence rate increase during the pandemic. In contrast to other studies, we report results for almost the first two years of the pandemic in a large tertiary and referral center. Better adherence to infection prevention protocols, decreased length of stay, and decreased hospital occupancy may have been prominent early in the pandemic leading to a decrease in CDI incidence. However, the current study analyzed a greater period and revealed significantly higher patient-days numbers during the pandemic, even though a drop in the hospital’s patient-days was indeed noticed during the first two pandemic months.
